# MIF and CXCL12 in Cardiovascular Diseases: Functional Differences and Similarities

**DOI:** 10.3389/fimmu.2015.00373

**Published:** 2015-07-21

**Authors:** Emiel P. C. van der Vorst, Yvonne Döring, Christian Weber

**Affiliations:** ^1^Institute for Cardiovascular Prevention, Ludwig-Maximilians-University Munich, Munich, Germany; ^2^German Centre for Cardiovascular Research (DZHK), Partner Site Munich Heart Alliance, Munich, Germany; ^3^Cardiovascular Research Institute Maastricht (CARIM), Maastricht University, Maastricht, Netherlands

**Keywords:** cardiovascular disease, atherosclerosis, chemokines, macrophage migration-inhibitory factor, CXCL12

## Abstract

Coronary artery disease (CAD) as part of the cardiovascular diseases is a pathology caused by atherosclerosis, a chronic inflammatory disease of the vessel wall characterized by a massive invasion of lipids and inflammatory cells into the inner vessel layer (intima) leading to the formation of atherosclerotic lesions; their constant growth may cause complications such as flow-limiting stenosis and plaque rupture, the latter triggering vessel occlusion through thrombus formation. Pathophysiology of CAD is complex and over the last years many players have entered the picture. One of the latter being chemokines (small 8–12 kDa cytokines) and their receptors, known to orchestrate cell chemotaxis and arrest. Here, we will focus on the chemokine CXCL12, also known as stromal cell-derived factor 1 (SDF-1) and the chemokine-like function chemokine, macrophage migration-inhibitory factor (MIF). Both are ubiquitously expressed and highly conserved proteins and play an important role in cell homeostasis, recruitment, and arrest through binding to their corresponding chemokine receptors CXCR4 (CXCL12 and MIF), ACKR3 (CXCL12), and CXCR2 (MIF). In addition, MIF also binds to the receptor CD44 and the co-receptor CD74. CXCL12 has mostly been studied for its crucial role in the homing of (hematopoietic) progenitor cells in the bone marrow and their mobilization into the periphery. In contrast to CXCL12, MIF is secreted in response to diverse inflammatory stimuli, and has been associated with a clear pro-inflammatory and pro-atherogenic role in multiple studies of patients and animal models. Ongoing research on CXCL12 points at a protective function of this chemokine in atherosclerotic lesion development. This review will focus on the role of CXCL12 and MIF and their differences and similarities in CAD of high risk patients.

## Introduction

Worldwide, cardiovascular disease (CVD) is the leading cause of death, accounting for more than 15 million deaths annually (1, 2). CVD is a collection of various diseases, but the most common and most severe are coronary artery disease (CAD) and cerebrovascular disease. In a high percentage of patients, these diseases eventually result in a myocardial infarction (MI) or stroke, respectively. CVD has not only a major impact on personal health, but also the economic burden is quite high. Besides the high mortality rates, patients who do survive are often hospitalized or should receive lifelong treatment, leading to high healthcare costs. In the United States alone, these costs are even more than $312 billion per year, indicating the magnitude of economic burden caused by CVD (3). Finally, the social burden on the direct environment of CVD patients should not be underestimated.

In recent years, or even decades a lot of research has been performed to better understand the exact pathology behind CVDs. Currently, atherosclerosis, a chronic inflammatory disease mainly affecting medium and large-sized arteries, has been identified as the main underlying cause of CVD (4). Upon activation of the vascular endothelial layer, lipids, immune cells, and cell debris start to accumulate in the vessel wall, forming initial lesions. These lesions will over time progress and grow in size, thereby partially or even fully occluding the artery. More often, however, the full occlusion of the vessel is caused by a rupture of the atherosclerotic lesion resulting in thrombus formation (4). Excessive growth or rupture of the lesion both result in ischemic areas in downstream tissues. Most commonly this occurs in arteries from the heart or the brain, leading to MI or stroke, respectively. To date, there is still no absolute suitable therapy available to cure or reverse atherosclerosis. Better understanding of this pathology will increase the ability to prevent it, and create opportunities to develop therapeutic strategies to cure it, or at least slow down the disease progression.

**Figure 1:**
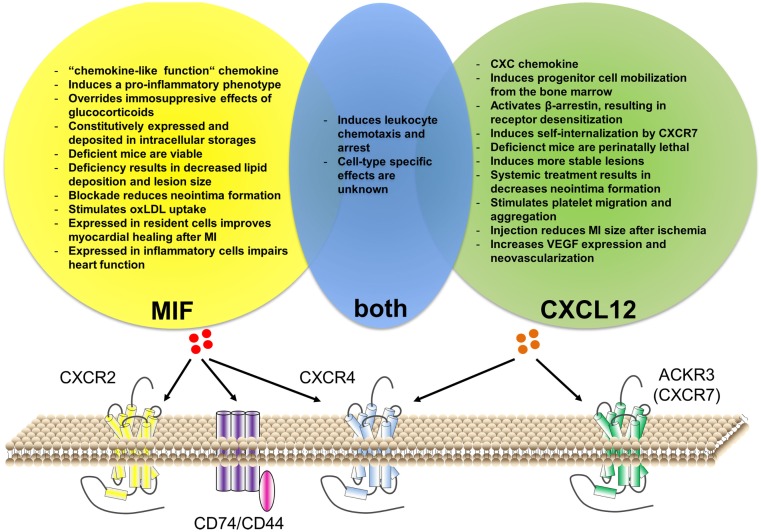
**Similarities and differences of CXCL12 versus MIF function in cardiovascular disease**. Both CXCL12 and MIF can bind to CXCR4. Additionally, CXCL12 can bind to ACKR3 (CXCR7), while MIF binds to CXCR2 and CD74/CD44. Although MIF interaction with ACKR3 has been described for platelets, it is still unclear whether this is via direct binding or via receptor heterodimerization. Both chemokines have an important role in leukocyte recruitment, although cell-type-specific effects remain unknown. Besides this, CXCL12 and MIF have individual functions associated with cardiovascular disease.

This review will give a short overview of the current knowledge about atherosclerosis, mainly focusing on the inflammatory aspects and the role of chemokines. Subsequently, the role of two important inflammatory mediators that recently have been connected with CVD and atherosclerosis will be discussed and put into clinical perspective, namely the chemokines CXCL12 and macrophage migration-inhibitory factor (MIF, Table 1 and Figure 1).

**Table 1:** **Overview of human studies on CXCL12 and MIF in CVD**.

Study design	Outcome	Reference
**CXCL12**		
Genome-wide association studies in over 100,000 patients	CXCL12 locus on chromosome 10q11 is clearly associated with CAD, indicating that CXCL12 may be involved in CVD development	(5)
Western blot analysis and immunohistochemical analysis of human plaques	Atherosclerotic lesions express high levels of CXCL12, in contrast to vascular cells of healthy vessels, associating CXCL12 with CVD	(6)
Cohort study of 904 patients with CAD	Platelet CXCL12 expression is increased in angina patients, though clinical significance remains to be elucidated	(7)
Cohort study of 215 patients with symptomatic CAD undergoing percutaneous coronary intervention	CXCR4 and ACKR3 are more highly expressed on platelets from CAD patients, associating receptors of CXCL12 to CVD	(8)
Plasma CXCL12 evaluation of 60 CAD patients	Plasma CXCL12 levels and surface expression of CXCR4 in peripheral blood mononuclear cells are decreased in angina patients, indicating that CXCL12 could be beneficial for CVD	(9)
Cohort study of 785 patients undergoing angiography	Plasma CXCL12 levels are superior to traditional risk factors in predicting CAD outcomes	(10)
Evaluation of 1,000 patients hospitalized due to chest pain	Platelet-derived CXCL12 expression occurs fast after injury in CAD patients, as early as 30 min, indicating that CXCL12 might be very useful as biomarker	(11)
**MIF**		
Single nucleotide polymorphism (SNP) evaluation of 459 MI patients and healthy controls	MIF single nucleotide polymorphism (rs755622) is associated with MI risk	(12)
MIF analysis in healthy and diseased internal mammary arteries	MIF is abundantly expressed in human atherosclerotic lesions, throughout lesion development, associating MIF with CVD	(13)
Immunohistochemical analysis of human atherosclerotic plaques	MIF is associated with fibrous cap weakening, by inducing protease expression and activity, associating MIF with plaque instability	(14)
Evaluation of 286 patients with symptomatic CAD undergoing percutaneous coronary intervention	Plasma MIF levels are increased in CVD patients, associated with inflammatory marker expression	(15)
Prospective study of 617 patients with CAD	High plasma MIF levels are an independent risk factor for future coronary events in CVD patients with impaired glucose tolerance or type 2 diabetes mellitus, associating MIF with CVD development	(16)
Plasma MIF and Grem 1 evaluation in 286 patients with CVD	High plasma Grem1/MIF ratio is associated with CVD and the grade of plaque stability, indicating MIF as a possible novel risk marker in CVD patients	(17)
Evaluation of MIF levels in patients with chronic stable angina	MI patients have higher plasma MIF levels which are predictive of final infarct size and remodeling, suggesting a role for MIF as biomarker	(18)
Prospective case–control study nested in the EPIC-Norfolk cohort in people without prior history of MI or stroke	Association of MIF with MI risk or death due to CVD is not very strong in humans without prior history of CVD, indicating that more research is necessary before choosing MIF as therapeutic target	(19)

## Atherosclerosis

### Risk factors

Already a multitude of risk factors have been described for CVDs, mainly derived from epidemiological studies. Main risk factors include psychosocial factors, hypertension, changes in low-density lipoproteins (LDL) and high-density lipoproteins (HDL) cholesterol levels, physical inactivity, smoking, obesity, lack of fruits and vegetables consumption, and alcohol and diabetes mellitus (20–22). All these risk factors vary greatly in prevalence and potency to influence CVD and are often combined in patients with severe atherosclerosis, supporting the concept that atherosclerosis is a multifactorial, complex disease (22). A worldwide case-control study showed that these nine factors combined account for more than 90% of the cardiovascular risk (21), where smoking and abnormal lipids already account for two-thirds of the risk. Additionally, many patients, especially high risk patients, have genetic predispositions for atherosclerosis (23). Some of these risk factors should be relatively easy to prevent with life-style adjustments. However, due to the multifactorial characteristics of the disease, identification of the precise mechanisms of action, whereby these risk factors influence atherosclerosis, is a key and crucial step in the road toward therapeutic strategies. This way for example statins, lipid modulating drugs, have been developed that lower LDL cholesterol levels and thereby inhibit the progression of atherosclerosis. Recently, Nahrendorf and Swirski very nicely reviewed the possible influence of various risk factors on the crosstalk between hematopoiesis, immune cells, and the cardiovascular system (22).

### Atherosclerosis initiation

Formation of atherosclerotic lesions occurs predominantly at predisposed sites, i.e., sites of disturbed laminar flow, like bifurcations and curvatures (24), thereby disturbing the normal, quiescent state of the endothelium. The resulting increased permeability of the endothelial layer leads to the accumulation of lipids, more specifically LDL, in the subendothelial layer of the arterial wall (25). LDL is very susceptible to oxidation, mainly caused by myeloperoxidase, lipoxygenenases or by reactive oxygen species such as HOCl, resulting in oxidized-LDL particles (oxLDL) (26). The modification of LDL can activate the endothelial cells (ECs) and tissue-resident macrophages (27). Subendothelial/extravasated LDL will be oxidized and taken up by resident macrophages via scavenger receptors (SRs), mainly SR-A, SR-B1, and cluster of differentiation (CD) 36 (28, 29). Intracellularly, oxLDL will be hydrolyzed into free cholesterol and fatty acids in late endosomes (30). Free cholesterol is subsequently transported to the endoplasmic reticulum where it undergoes re-esterification to cholesteryl esters by the acyl-CoA:cholesterol ester transferase (ACAT) enzyme (31). This accumulation of cholesteryl esters will transform the macrophages into foam cells, a characteristic hallmark of early atherosclerosis. Not only the uptake and storage of cholesterol are disturbed, but also the excretion mechanisms are disturbed. ATP binding cassette transporters A1 and G1 (ABCA1 and ABCG1) are the major contributors to this cholesterol efflux through reverse cholesterol transport (RCT) (32, 33). Cholesterol will efflux toward HDL, making HDL beneficial for atherosclerosis development (32). Normally, this efflux mechanism is upregulated upon lipid loading, however, during hypercholesterolemia this route is compromised via TLR4-induced downregulation of the cholesterol transporters, further favoring the conversion of macrophages to foam cells (34).

This macrophage activation, due to the lipid loading, together with the activation of ECs will lead to more vascular inflammation, by the secretion of various cytokines, chemokines, and adhesion molecules (35). The inflammation will result in the attraction of mainly monocytes but also other immune cells, like T- and B-lymphocytes and neutrophils to the site of injury and lesion formation (26). In humans, two main subsets of circulating monocytes have been described based on their expression of CD14 and CD16 (36). Over 90% of the circulating monocytes are CD14^high^/CD16^−^ and are called the classical monocytes (37). The other population, the non-classical monocytes are CD14^low^/CD16^high^ (36). These distinct subsets also show differential expression of chemokine receptors (38). The classical monocytes express, for example, high levels of CCR2, a receptor for CCL2 (39). CX_3_CR1 is expressed on both subtypes, but the expression on non-classical monocytes is twofold higher compared to that on the classical monocytes (40, 41). In mice, also two main subtypes of circulating monocytes can be identified, discriminated mainly by the expression of Ly6C, i.e., the Ly6C^high^ (classical) and Ly6C^low^ (non-classical) monocytes (42). Similar to the human subpopulations mouse classical monocytes express high levels of CCR2, while non-classical monocytes express CX_3_CR1 (43). Hypercholesterolemia, a common characteristic of atherosclerosis increases Ly6C^high^ monocyte levels (44, 45). Additionally, Ly6C^high^ classical monocytes form the main subtype that will migrate to the site of injury (46).

The mechanism of monocyte recruitment and adhesion consists of complex interactions between various adhesion molecules and chemokines. The exact role of various chemokines in atherosclerosis will be discussed later. In short, the monocyte adhesion cascade consists of several steps. The first step in this cascade is the capture and rolling phase, where various chemokines and selectins on the luminal side of the activated endothelium play a crucial role (35, 47). The second step is the firm adhesion of these monocytes to the endothelium. In this phase, vascular cell adhesion molecule 1 (VCAM1) and intercellular adhesion molecule 1 (ICAM1) present on the endothelium are essential, since they attach firmly to the integrins very late antigen 4 (VLA4) and lymphocyte function-associated antigen 1 (LFA1), respectively, present on the monocytes (47). VCAM1, ICAM1, and E-selectin form clusters to initiate cytoskeletal reorganization, necessary for subsequent transmigration (48). These clusters, also called docking structures, have been shown to be located on upright endothelial membrane processes and are organizes as the so-called transmigratory cups by tetraspanins (49). Monocyte transmigration across the endothelial layer is mainly directed by various chemokines and their receptors (50). Next to these chemokines, also the endothelial junction molecules, platelet endothelial cell adhesion molecule 1 (PECAM1), VE-Cadherin, and junctional adhesion molecules (JAMs) play a crucial role as regulators of EC permeability and leukocyte transmigration (51). Monocytes have two ways to transmigrate, a paracellular route and a transcellular route. The paracellular route leads monocytes through the endothelial junctions, whereas the transcellular route uses fusing vesicles in the endothelium cell cytoplasm for transmigration (52). Once inside the vessel wall, monocytes can differentiate into macrophages, driven by macrophage colony-stimulating factor (M-CSF) (53). Macrophages are a heterogeneous cell population, consisting of two main groups, the classically activated, inflammatory M1 macrophages and the alternatively activated, inflammation resolving M2 macrophages (54). Various cytokines play a role in this polarization of macrophages, such as IL-10 and interferon-γ (55). Both types of macrophages are present in atherosclerotic lesions, where the balance between them is of great importance for either plaque development or resolving inflammation (56). These attracted leukocytes will again be exposed to the oxidized-lipid-rich environment of the developing lesion, forming foam cells. Thereby a vicious circle of leukocyte attraction and lipid accumulation is formed, stimulating atherosclerosis development.

### Plaque progression

When lesional macrophages take up so many lipids and debris, many will eventually go into apoptosis. In early plaque development apoptosis is not considered detrimental, since neighboring macrophages will take up and eliminate the apoptotic debris, a process called efferocytosis (56). However, when plaque development progresses, the excessive uptake of lipids and debris continues and eventually leads to cellular stress and impaired efferocytosis (57). This will result in the accumulation of apoptotic debris and apoptotic macrophages will go into secondary necrosis, leading to the formation of the necrotic core which is characteristic of more advanced lesions (56, 58). The necrotic core will significantly contribute to the lesional inflammation, and thus progression, and also contains pro-thrombotic factors that will lead to a thrombus when it comes into contact with platelets (59). To prevent this from happening, a fibrous cap is formed between the necrotic core and the lumen, by deposition of mainly collagen and elastin by intimal smooth muscle cells (SMCs) (60). Various cytokines and growth factors, produced by leukocytes, are important for the migration of intimal SMCs to the intima and for the extracellular matrix production (60). Plaques with a big fibrous cap are considered to be more stable atherosclerotic lesions, i.e., less prone to rupture. However, macrophages can also produce matrix metalloproteinases (MMPs), which are capable of degrading extracellular matrix proteins (59). Fibrous cap degradation and thus thinning makes the lesion more vulnerable and can eventually lead to a plaque rupture, releasing pro-thrombotic material into the bloodstream resulting in thrombus formation and obstruction of blood flow. This can cause ischemia to distal regions and result in a MI or stroke (58).

### Chemokines in atherosclerosis

Chemokines are the largest family of cytokines, consisting of small molecules (8–12 kDa) that exert chemotactic effects on cells (61). This large family is divided into four subclasses, being C, CC, CXC, and CX_3_C. This classification is based on the position of the N-terminal cysteine residues (62). In addition to these four canonical chemokine classes, a fifth subclass consisting of molecules that share functional similarities with chemokines has emerged, referred to as “chemokine-like function” (CLF) chemokines (63). These non-canonical chemokines do exert some CLFs, but do not contain the specific N-terminal cysteine residue characteristic of canonical chemokines (63). Chemokines can bind to chemokine receptors, which are classified according to the chemokine they bind. Most chemokine receptors will activate G proteins and intracellular signaling upon binding and are therefore part of the G protein-coupled receptor family. However, several receptors, the so-called atypical chemokine receptors, are G protein signaling independent and rather play a role in the scavenging of chemokines (64). Chemokines and chemokine receptors are expressed on various cell types, like ECs, SMCs, and leukocytes. Originally, chemokines were discovered for their capacity of directing leukocytes toward sites of inflammation (65). Thereby, chemokines also play a crucial role in atherosclerosis.

Chemokines already play a role in a very early stage of atherosclerosis development. Recently, it was shown that lysophosphatidic acid, a component of LDL mediates the release of CXCL1 from ECs (66). Studies in atherosclerotic prone ApoE^−/−^ mice fed a cholesterol-rich diet show that CXCL1 is not only important for the mobilization of the classical, or inflammatory, monocytes to the site of inflammation, but also important for neutrophil recruitment via the receptor CXCR2 (66–68).

The involvement of various chemokines and their receptors in monocyte recruitment has already been well described (69). However, during the recent decade these described involvements have been revised and are still being greatly debated. Using a highly sophisticated technique of transferring atherosclerotic aortic arches from ApoE^−/−^ mice into specific chemokine receptor knockout mice, it was shown that inflammatory monocytes require CCR2, CCR5, and CX_3_CR1 to migrate into the atherosclerotic lesion, while the patrolling monocytes used CCR5 for recruitment (70). However, this view again changed recently using adoptive transfer experiments with pharmacological inhibition of the specific chemokine receptors. Here it was shown that the inflammatory Ly6C^high^ monocytes use CCR1 and CCR5 for recruitment, rather than the previously shown CCR2 and CX_3_CR1 (68). CCR2-deficient mice on an atherosclerosis-prone background did, however, result in a significantly reduced lesion size in mice. Based on the adoptive transfer study and the fact that CCR2-deficient mice show reduced circulating monocyte counts, it can be suggested that the beneficial effect on atherosclerosis is due to its effects on monocyte release from the bone marrow, rather than directs effects on monocyte recruitment (71, 72). Also for CX_3_CR1-deficient mice, it was observed that these animals have reduced atherosclerosis development, implicating CX_3_CR1 in atherogenesis (73). However, instead of effects on monocyte recruitment, CX_3_CR1 seemed to play an important role in cell survival. Thus, deficiency of this chemokine resulted in increased apoptosis of plaque macrophages, thereby reducing lesion development (73). CCR1 and CCR5, which do seem to be involved in leukocyte recruitment, both have several specific ligands, but also share ligands like CCL3 and CCL5. However, looking at the exact effects of both receptors on atherogenesis, there are distinct differences. CCR5 deficiency results in a clear reduction of diet-induced atherosclerosis in mice, while CCR1 deficiency increased lesion development (74, 75).

In the later stages of atherosclerotic lesion development, chemokines also still play an important role. The best-studied chemokine receptor in this stage, especially with respect to plaque regression, is CCR7. It has been shown in various studies that CCR7 is necessary for the egress of macrophages during lesion regression (76–78). However, CCR7 in T cells seems to play a pro-atherogenic role. CCR7-deficient T cells show an impaired entry and exit capacity from atherosclerotic lesions (79). Combined, CCR7 is thus involved in macrophage egress from lesions and T cell migration. In these later stages of lesion development also CXCL10 is crucially involved, especially in plaque stability. Inhibition of CXCL10 resulted in relatively more SMCs and a more stable plaque phenotype (80).

These results clearly show that the chemokine system plays an important role in all stages of atherosclerotic lesion development, but underlines that these interactions are very complex and elaborate. Additional research is still needed to even further elucidate the role of this system in atherosclerosis and CVD in general. Besides the already described chemokines involved in atherosclerosis, in the recent years, more and more research has been focusing on two yet undiscussed chemokines, being CXCL12 and MIF. The remainder of this review will specifically focus on the role of these two chemokines in atherosclerosis and CVD.

## CXCL12

### Ligand/receptor characteristics

CXCL12, also known as stromal cell-derived factor 1 (SDF-1), is one of the 17 members of the CXC chemokine family (5). Structurally, this group of chemokines can be further subdivided into two groups, depending on the presence of a specific amino acid motif [glutamic acid–leucine–arginine (ELR)] before the first cysteine group (81). Interestingly, this subdivision showed also to be a functional separation since ELR-positive chemokines attract neutrophils, while ELR-negative chemokines, such as CXCL12, attract T lymphocytes and natural killer cells (82, 83). CXCL12 consists on its turn again of six isoforms, derived from alternative splicing (84). The classical isoforms are CXCL12-α and CXCL12-β, which are expressed throughout the body and so far functionally indistinguishable (84). Other isoforms are called CXCL12-γ, -δ, -ε, and -φ, which show a more restricted expression pattern and are until now much less studied. All isoforms share the same N-terminal sequence, but differ in the C-terminal region (84).

The most important receptor for CXCL12 is CXCR4, which is also expressed on a wide variety of cells (85, 86). CXCR4 is a G protein-coupled receptor and binding of CXCL12 will induce intracellular signaling via a classical heterotrimeric G protein (86). Receptor activation has been shown to trigger MAPK and PI3K signaling, but also calcium mobilization (87). Additionally, activation of CXCR4 results in β-arrestin recruitment, resulting in the endocytosis of CXCR4 and thus receptor desensitization (88).

More recently, a second receptor for CXCL12 was identified, being ACKR3 (CXCR7) which is highly expressed on monocytes and mature B cells (89). This receptor has even a 10-fold higher affinity for CXCL12 than CXCR4. Binding of CXCL12 to the receptor does, however, not result in the classical leukocyte chemotaxis response or coupling with G proteins to induce intracellular signaling. ACKR3 is implicated in cell growth and survival (90). However, the main function of ACKR3 seems to be as a decoy receptor, since receptor stimulation by CXCL12 enhances internalization of ACKR3 and thereby delivery of its ligands to the lysosomes for degradation (91, 92). This way, ACKR3 activation would reduce CXCL12/CXCR4 signaling. However, since CXCL12 scavenging also prevents the downregulation of CXCR4, ACKR3 could also well be beneficially influencing CXCR4-mediated effects (93). However, ACKR3 stimulation has also been shown to result in downregulation of CXCR4 (94). On the other hand, CXCR4 seems also to be influencing ACKR3, since the widely used antagonist for CXCR4 AMD3100 has recently been shown to have agonistic effects on ACKR3 (95). ACKR3 can have intracellular signaling effects on MAPK, but these are purely β-arrestin mediated (96). Altogether, these results clearly show that there is a complex interaction between CXCL12/CXCR4 and ACKR3.

### CXCL12 and stem-/progenitor-cell mobilization

The importance of CXCL12 in general has been clearly shown in mice that have a total CXCL12 deficiency. These animals die already perinatally due to major defects in hematopoiesis, vasculo-, cardio-, and neurogenesis (97). These embryonic defects are indicative for the important role of the CXCL12/CXCR4 axis in progenitor cell migration, but also for survival and chemotaxis of murine embryonic stem cells during embryogenesis (98). The role of CXCL12/CXCR4 in this mobilization from the bone marrow has already been well studied, not only for hematopoietic stem cells, but also for EC and SMC progenitor cells (99). In physiological conditions, hematopoietic stem cells are retained in the bone marrow by high expression of CXCL12 by stromal cells (97). In the clinic, modulation of the CXCL12/CXCR4 axis by granulocyte colony-stimulating factor (G-CSF) is already used to mobilize stem cells from the bone marrow to the circulation. G-CSF has various ways of modulating this axis, reviewed in (97). Additionally, the role of CXCL12/CXCR4 is confirmed by using the CXCR4 antagonist AMD3100, which results in decreased bone marrow CXCL12 levels, thereby favoring mobilization of stem cells (100).

### CXCL12 in atherosclerosis

Mobilization of hematopoietic stem cells, but also progenitor cells like endothelial progenitor cells (EPCs) and smooth muscle progenitor cells (SPCs), has also been implicated in various pathologies like atherosclerosis (101, 102). A recent study showed that injections of CXCL12 in mice developing atherosclerosis resulted in more stable lesions, characterized by more SMCs and a thicker fibrous cap (103). These plaque-stabilizing effects were mediated by an increased recruitment of SPCs to these lesions. Supporting this finding, direct injection of SPCs in mice reduces atherosclerotic lesion development and improves the stability (104). Besides SPCs, also EPCs were shown to be involved in atherogenesis, since infusion of EPCs, or AMD3100 treatment triggering EPC mobilization resulted in a beneficial effect on lesion regression (105). Together, these studies show atheroprotective effects of the CXCL12/CXCR4 axis, mediated by progenitor mobilization.

Besides these effects on progenitor cells, mediating beneficial effects on atherosclerosis, CXCL12/CXCR4 may also influence disease development by influencing various atherosclerosis-related cells. This is especially interesting, since CXCR4 is expressed on basically every cell-type related to atherogenesis, like monocytes, macrophages, neutrophils, ECs, SMCs, and T- and B-cells (106–110). However, until now there have been no studies directly investigating the causal cell-type-specific effects of CXCL12/CXCR4 in relation to atherosclerosis. Though there are already various studies at least associating this axis with atherogenesis.

For macrophages, it has been shown that oxLDL, also in large amounts present in atherosclerotic lesions, upregulated CXCR4 expression which could contribute to macrophage migration (111). The CXCL12/CXCR4 signaling in macrophages was also implicated in macropinocytosis, indicating a possible influence on lipid accumulation in these cells (112). Furthermore, it has also been associated with neutrophils as it regulates the release of neutrophils from the bone marrow (113). Not only the release is mediated by this axis, but also the clearance of circulating neutrophils as senescent neutrophils shows increased expression levels of CXCR4-mediating effective clearance (114). By contrast, activated neutrophils downregulate CXCR4 levels, leading to postponed clearance (97). Treatment of ApoE^−/−^ mice with AMD3100 resulted in an increased neutrophil mobilization, thereby increasing atherosclerotic lesion areas (44, 45). ECs also release more CXCL12 after oxLDL stimulation (115). Additionally, laminar shear stress appeared to influence CXCR4 expression, where high shear suppresses CXCR4 expression (116). CXCL12 can also increase vascular endothelial growth factor (VEGF) expression in ECs, which promotes angiogenesis (117). As angiogenesis is inducing a more vulnerable plaque phenotype, CXCL12 could have lesion destabilization effects. Chemotaxis of both T- and B-cell is also positively influenced by CXCL12/CXCR4 (118–121). Besides CXCL12/CXC4 effect on all these different cell-types, expression of CXCL12/CXCR4 has also been shown on platelets (6, 122). Platelets are the first to arrive at a site of vascular injury, where its glycoproteins Ib and IIb/IIIa engage surface molecules on the ECs, contributing to endothelial activation (4). Platelets also produce and store CXCL12 in their α-granules. Upon release, platelet-derived CXCL12 has been implicated in cell adhesion and chemotaxis (123). Furthermore, CXCL12 is able to induce platelet aggregation, a crucial step in thrombus formation after atherosclerotic plaque rupture (6). Platelets also express the receptor CXCR4, and blocking studies indicated that this receptor is crucially involved in the aggregation effects of CXCL12 (6). Additionally, CXCL12 is able to stimulate platelet migration and transmigration (124). Together, these results show that CXCL12/CXCR4 have interactions with many cells that are relevant in atherosclerosis and is thereby modulating atherosclerosis development. Recently, also a role for ACKR3 in atherosclerosis development has been described, showing that activation of ACKR3 by a synthetic ligand reduced lesion formation and ameliorated hyperlipidemia. ACKR3 seemed to play an important role in the regulation of blood cholesterol levels, by promoting VLDL uptake in adipose tissue (125).

### CXCL12 in atherosclerosis-related pathologies

As previously described, atherosclerotic plaques eventually, either block an artery by growth or by rupture and subsequent thrombus formation. The result is ischemia in downstream tissues. One of the most common places for this to occur is in the heart, resulting in a MI. It is known that hypoxia results in an upregulation of CXCL12 and CXCR4 (126). Various studies have revealed a protective role for CXCL12/CXCR4 signaling after MI through survival effects on resident cardiomyocytes and recruitment of protective circulating cells (97). Direct injection of CXCL12, for example, reduced myocardial infarct size after ischemia, which was associated with increased neo-angiogenesis (127). Recruitment of progenitor cells was crucial for this improved vessel growth (128). Supporting a role for CXCL12/CXCR4 in MI are studies using AMD3100, an antagonist for CXCR4. However, results from these studies are quite contradictory. A study using a single injection of AMD3100 showed improved cardiac function and enhanced progenitor cell accumulation and neovascularization (129, 130). However, more chronic administration of AMD3100 showed reduced incorporation of progenitor cells and cardiac outcome after MI (131).

CXCL12/CXCR4 has also been shown to play a role in vascular restenosis. The main treatment of choice after arterial blockage is percutaneous coronary intervention, where a stent is placed at the site of lesion development. However, neointimal hyperplasia often causes restenosis of these stents mainly driven by SMCs, thereby again occluding the artery. Systemic treatment of mice that underwent wire-induced arterial injury with CXCL12 or CXCR4 antagonists showed clear reductions of neointimal size and SMC content (132, 133). This reduced SMC content was caused by a reduction in progenitor mobilization toward the site of injury (132, 133). Additionally, CXCR4 blockage reduced cellular proliferation at sites of neointimal lesions (134). A significant decrease in EPC mobilization was recently also observed using genetic EC-specific knockout of CXCR4, although mice showed larger neointimal lesions consisting of more inflammatory macrophages, but less SMCs (135).

### Human and clinical implications for CXCL12

Genome-wide association studies showed clear associations of CXCL12 with CVD (5) (Table 1). Previously, it was already shown that vascular cells express high levels of CXCL12 in human atherosclerotic lesions, but not in normal vessels (6). Various human studies supported the idea that CXCL12 is a potential regulatory agent in atherosclerosis. A study, comparing plasma CXCL12 levels of angina patients with healthy controls, showed decreased levels of CXCL12 in the patient group (9). Patients with unstable angina had even lower CXCL12 levels than those with stable angina. Additionally, these patients showed decreased surface protein expression of CXCR4 in peripheral blood mononuclear cells, while the RNA expression was increased probably as compensatory mechanism (9). Combined, this study suggests anti-atherogenic properties of CXCL12. In contrast, another study observed significantly increased CXCL12 expression on platelets of stable angina patients, compared to healthy controls. Plasma CXCL12 levels also correlated with platelet activation (7), suggesting a more atherogenic and pro-thrombotic role for CXCL12. In addition, both CXCR4 and ACKR3 are more highly expressed on platelets from CVD patients, compared to healthy controls (8). It is suggested that platelet-derived CXCL12 expression occurs relatively fast after vessel injury, being as early as 30 min (136). Currently, used biomarkers for acute coronary syndrome, like troponin-I are much slower. Therefore determination of CXCL12 might be very useful as early additional biomarker (11). Recently, it was shown that CXCL12 is also associated with heart failure (137), and that plasma CXCL12 levels are even superior to ­traditional risk factors in predicting adverse cardiovascular outcomes (10). Although there are still some contradictory clinical results, it is clear that CXCL12 does play a role in atherosclerosis and CVD.

## Macrophage Migration-Inhibitory Factor

### Ligand/receptor characteristics

Discovered almost 50 years ago, macrophage MIF was one of the first cytokines to be identified (138). MIF is part of the CLF chemokine family, as it is missing the typical N-terminal cysteines (139). The name is derived from its discovery as MIF-containing supernatant showed to be inhibitory for macrophage migration (140). At first, T cells were thought to be the main cellular source of MIF. However, since its discovery, expression of MIF has also been shown in other immunity cells like monocytes, macrophages, neutrophils, dendritic cells, and B cells (13, 141–146). In contrast to many other chemokines, MIF is constitutively expressed and deposited in intracellular storages. Thus, upon stimulation, MIF release does not require *de novo* synthesis (138). It has already been well described that MIF can directly or indirectly stimulate a large variety of pro-inflammatory molecules, including various cytokines and nitric oxide. Additionally, MIF was shown to override the immunosuppressive effects of glucocorticoids (147). MIF has been implicated in various acute and chronic inflammatory diseases, like sepsis, rheumatoid arthritis, and cancer (148–150).

The first receptor identified for MIF was CD74, the membrane-expressed form of invariant chain and an MHC class II chaperone (151). However, besides its role in antigenic peptide loading, CD74 can also be expressed in the absence of the MHC class II protein, thus exerting functions as membrane receptor (152). MIF binds with high affinity to CD74, although CD74 by itself is not able to induce intracellular signaling. Therefore, it requires the recruitment of signaling-competent co-receptors. CD44 was the first described co-receptor of CD74, able to mediate signal transduction (153). CXCR2 and CXCR4 have also been described as co-receptors for CD74 (154, 155). The combination of CD74 with CD44 has been linked with MIF’s pro-inflammatory and anti-apoptotic functions by the activation of MAPKs (153, 156). CD74/CXCR2 complexes have been shown to be involved in MIF-mediated monocyte chemotaxis and arrest. In line, a role for CD74 in atherogenesis has been identified (157).

CXCR4 has already been discussed previously as receptor for CXCL12. It has been found, in monocytes, T cells and fibroblasts, that CXCR4 can also form heterodimers with CD74 and induce Akt signaling (155). CXCR4 has mainly been shown as the receptor responsible for MIF-induced T cell recruitment (154). Finally, CXCR2 has been described as important receptor for MIF. MIF/CXCR2 interaction mainly triggered the recruitment and arrest of monocytes. Furthermore, MIF/CXCR2 has been implicated in integrin activation, an important step in leukocyte recruitment. Recently, ACKR3 on platelets has also been described as receptor for MIF, although it is still not clear whether this is a direct ligand–receptor interaction or indirect interaction via receptor heterodimerization such as CXCR2/ACKR3 (158).

### MIF in atherosclerosis

Hyperlipidemia is one of the hallmarks of atherogenesis. It was shown that upon hyperlipidemia, MIF expression is greatly enhanced in cells crucial for atherosclerosis development, like ECs, SMCs, monocytes, and T cells (13, 159, 160). As atherosclerotic lesions progressed, MIF expression was even further increased. Combined, these data clearly implicate MIF not only in atherosclerotic lesion development, but also in plaque destabilization.

Leukocyte recruitment into atherosclerotic plaques is one of the most important processes during lesion development. *In vitro* adhesion assays under flow clearly showed an increased monocyte arrest of monocytes to aortic ECs upon MIF incubation (161). This was confirmed by using MIF neutralizing antibodies, which blocked the observed effects. Additionally, using small interfering RNA to inhibit endothelial MIF production, it was observed that MIF deficiency resulted in a decreased expression of E-selectin, ICAM-1, VCAM1, IL-8, and MCP-1, all important mediators of leukocyte recruitment (162). Bernhagen et al. clearly showed that MIF can also more directly trigger monocyte, neutrophil, and T cell arrest and chemotaxis in an integrin-dependent manner (154). They further implicated the receptors for MIF in this process, since the integrin activation resulted in the triggering of G_αi_ activities of CXCR2 in monocytes and neutrophils and of CXCR4 in T cells. Additionally, CD74 also contributes to monocyte recruitment by interacting with CXCR2 (154).

Various functional animal studies confirmed the role of MIF in atherosclerosis development. MIF-deficient mice on an atherogenic background showed significantly reduced lipid deposition and lesion size compared to control animals (163). This was accompanied by a decreased lesion cell proliferation, especially of SMCs. Additionally, neutralizing MIF with specific monoclonal antibodies showed a reduced lesion size and especially reduced intimal inflammation (160). MIF blockage was even showed to induce regression of already established atherosclerotic lesions (154). Additionally, MIF stimulates the uptake of oxLDL by macrophages and is associated with the expression of proteases (163), which can contribute to the lesion destabilization properties of MIF. Recently, also an important role for platelet-derived MIF was described (164). MIF was even shown to have a stronger chemotactic activity than CXCL12 and substantially contributed to monocyte adhesion to an endothelial layer. Although in contrast to CXCL12 secretion, MIF secretion from platelets was much slower and did not enhance platelet activation (164).

Studies with CXCR2-deficient mice also identified important roles for CXCR2 in monocyte recruitment into atherosclerotic lesions, showing reduced lesion size and lesional macrophage content (165). In atherosclerosis studies with other CXCR2 ligands, like CXCL1 and CXCL8, deficiencies did not exceed half the effect of the receptor deficiency on atherogenesis, suggesting the presence of other ligands that play a crucial role (166). In 2007, MIF was identified as ligand for CXCR2 with pro-atherogenic capacities (154). Combined, all these studies clearly identify MIF and its receptors as an important mediator of leukocyte recruitment and atherosclerosis development.

### MIF in atherosclerosis-related pathologies

As described earlier, restenosis occurs frequently after stent implantation, leading to early stent failure. Carotid artery wire injury methods are often used to model this disease, characterized by neointimal hyperplasia driven by SMC proliferation. Carotid artery injury resulted in a fast induction of MIF expression in SMCs and later on also in foam cell formation (161, 167). To determine the causal role of MIF in neointimal hyperplasia, antibody-mediated MIF blockage has been used in various studies. MIF blockage indeed resulted in a decreased medial cell proliferation, enhanced apoptosis, and smaller inflammatory cell content (167). Another study also showed a decreased macrophage content and foam cell formation upon MIF blockage (161). This was accompanied by an increase in SMC and collagen content in the neointimal areas, suggesting a more stable phenotype after MIF blockage (168).

Various studies also describe MIF as a protective factor in MI-ischemia-reperfusion injury (63). However, recently it has been shown that this effect is dependent on the cellular source of MIF. Global MIF deficiency protects the heart from post-infarct cardiac rupture and remodeling, by suppressing the leukocyte infiltration and thus inflammation (169). However, leukocyte-derived MIF exerts opposing effects by promoting the inflammatory response after MI (169). These compartmentalized and opposing effects are shown to be mediated by CXCR2 (170).

### Human and clinical implications for MIF

In humans, MIF has been shown to be abundantly produced by various cells in different stages of plaque development, indicating an important role for MIF in early plaque development but also in more advanced complicated lesions (13) (Table 1). Later, it was observed in human lesions that MIF plays a more important role in vulnerable lesions, compared to fibrous lesions. MIF was associated with the weakening of the fibrous cap, by inducing MMP-1 expression and activity in SMCs (14). CVD patients, more particularly patients with acute coronary syndromes also showed enhanced plasma MIF levels. Plasma MIF levels from these patients were associated with inflammatory markers like CRP and IL-6, but also with the cardiac necrosis marker troponin-I (15). High plasma MIF levels have also been identified as an independent risk factor for future coronary events in patients with CVD and impaired glucose tolerance or type 2 diabetes mellitus (16). Lately, the Grem1/MIF ratio has been identified as novel marker associated with CVD and the grade of plaque stability. Grem1 was discovered as an endogenous inhibitor of MIF (17). Furthermore, human epidemiological studies supported a pro-atherogenic role of MIF, by showing that a MIF single nucleotide polymorphism was associated with an enhanced risk for MI (12). A large group of MI patients also had elevated MIF plasma levels and these levels were predictive of final infarct size and the extent of cardiac remodeling (18). Elevated MIF levels in these patients were already observed after 4 to 6 h after acute MI, which could be very beneficial for the early detection of MI since current used markers or only elevated after 6 to 12 h post-MI (18).

There are already several MIF inhibitors developed, which show protective effects in various inflammatory models (168). Another attractive therapeutic strategy would be to directly target the receptors for MIF, CXCR2, or CXCR4, or to manipulate the ligand–receptor interaction. However, more research is first needed to fully elucidate these precise interactions. Although MIF seems like a suitable target for therapy and biomarker in patients, the use of MIF as biomarker in healthy persons should be approached with caution. Prospective data suggest that the relation between MIF and the risk of MI or death due to CVD in humans without prior history of CVD is not very strong (19).

### CXCL12 and MIF side by side

Both CXCL12 and MIF play an important role in the development of CVDs. However, besides some common effects both chemokines vary functionally from each other, partly mediated by differential receptor usage (Figure 1). An important common function that both chemokines have is the induction of leukocyte chemotaxis and arrest. However, as MIF seems to have more pro-atherosclerotic effects, CXCL12 may have a protective function, although results are still contradictory at some level and future research should further elucidate the exact role of these chemokines in atherosclerosis. Regarding vascular restenosis the effects of CXCL12 and MIF are more equal, since blockade studies showed that inhibition of either CXCL12 or MIF has beneficial outcomes on neointimal hyperplasia. Additionally, blockage of CXCR4, the receptor for both CXCL12 and MIF, has been shown to reduce neointimal formation. Furthermore, both CXCL12 and MIF play a protective role in MI-ischemia-reperfusion injury. For CXCL12, this beneficial effect upon systemic CXCL12 injection was associated with increased recruitment of progenitor cells and neo-angiogenesis. However, the beneficial effects of MIF seem to be cellular source dependent as global MIF deficiency reduced inflammation, while leukocyte-derived MIF promoted the inflammatory response after MI.

## Concluding Remarks

It has already been well described that chemokines play an important role in inflammation, atherosclerosis, and CVD. However, the exact involvement of all these chemokines remains very complicated and as research in this area advances, current ideas and dogmas may still change. In the recent years, more data are accumulating pointing toward crucial roles of these chemokines in atherosclerosis and CVD. There have also already been some studies describing the ligand–receptor interactions and the involvement of the receptors, CXCR2, CXCR4, and ACKR3 in different pathologies, although also in this respect further research is needed to identify cell-type-specific effects of CXCR4 for example, but also to clarify the triggered intracellular signaling. Due to the complexity of this chemokine system, one should be very cautious with designing chemokine-based therapeutics, since unwanted side effects may occur very easily. Therefore very specific targeting approaches, like antibodies or inhibitors, are needed to isolate a specific ligand–receptor interaction, perhaps even at a specific cell type.

## Author Contributions

EV: drafting the manuscript; YD: concept and design, critical revision; CW: concept and design, critical revision.

## Conflict of Interest Statement

The authors declare that the research was conducted in the absence of any commercial or financial relationships that could be construed as a potential conflict of interest.
